# Supplementary material for: A novel enhancer regulates MGMT expression and promotes temozolomide resistance in glioblastoma
Source: Nat Commun. 2018 Jul 27;9:2949. doi: 10.1038/s41467-018-05373-4 (PMC6063898; doi:10.1038/s41467-018-05373-4)
Supplement: Supplementary file 1 — Supplementary Information [file 41467_2018_5373_MOESM1_ESM.pdf]

Supplementary Materials for

A novel enhancer regulates MGMT expression and promotes temozolomide resistance in glioblastoma

Chen et al.

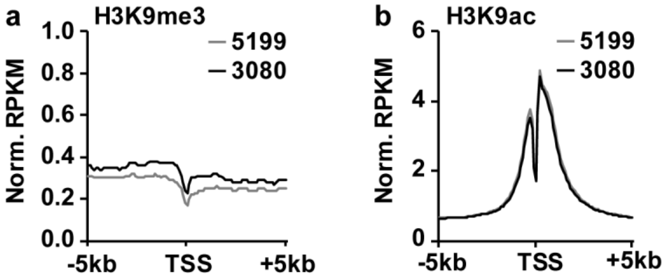

Supplementary Figure 1. Average levels of H3K9me3 and H3K9ac in GBM xenografts.

a-b. Aggregate plots showing the average ChIP-seq reads distribution of H3K9me3 (**a**) and H3K9ac (**b**) ChIP-seq reads surrounding transcription start site.

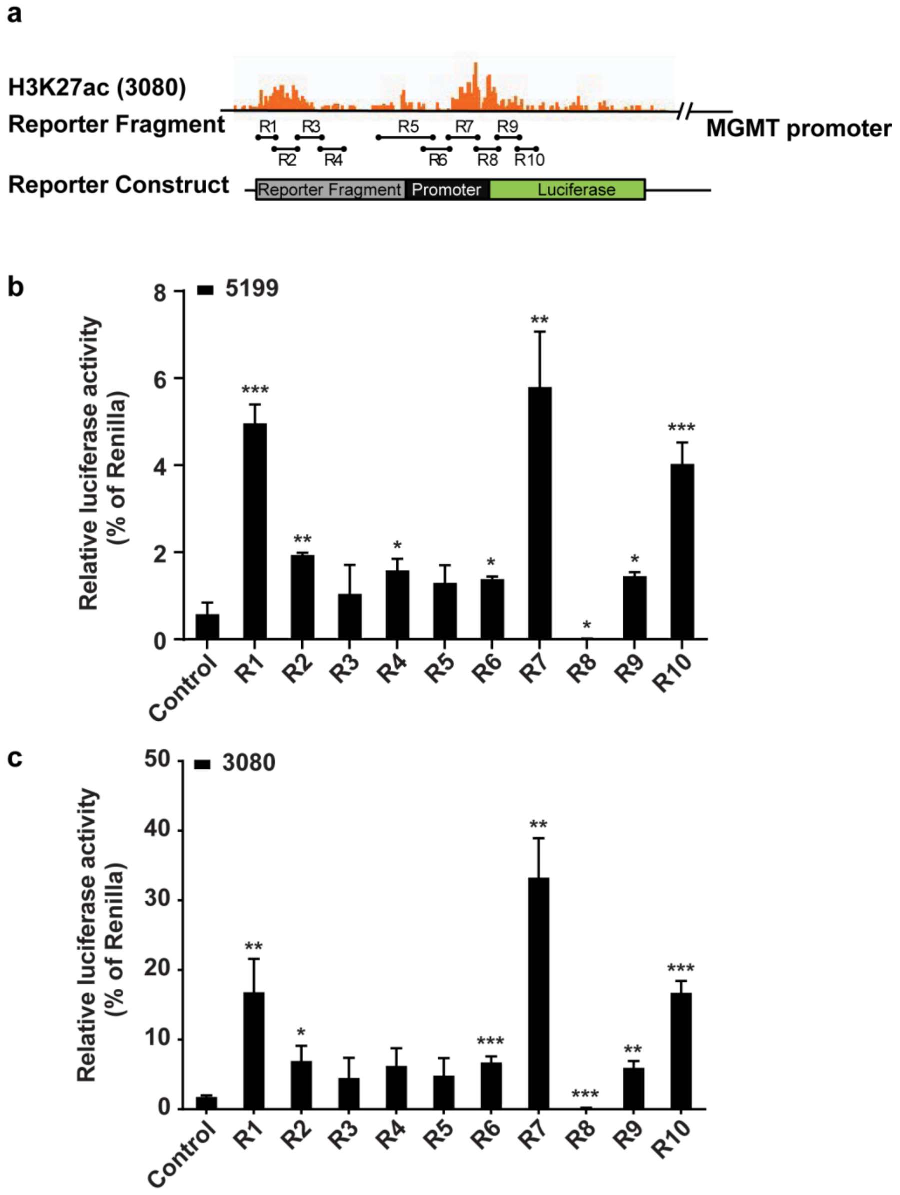

Supplementary Figure 2. Reporter assay read outs in 5199 and 3080 cells.

a. Schematic diagram showing the ten fragments (R1-R10) covering K-M enhancer region were cloned upstream of luciferase promoter. **b-c.** Effect of each fragment on transcription of luciferase was tested in 5199 (**b**) and 3080 (**c**) cells. Firefly and *Renilla* luciferase activity were measured 36h after transfection. Luciferase activity was normalized to the *Renilla* luciferase activity. Reported values were derived from 3 biological repeats. (*$p<0.05$, **$p<0.01$, ***$p<0.001$, student's *t* test)

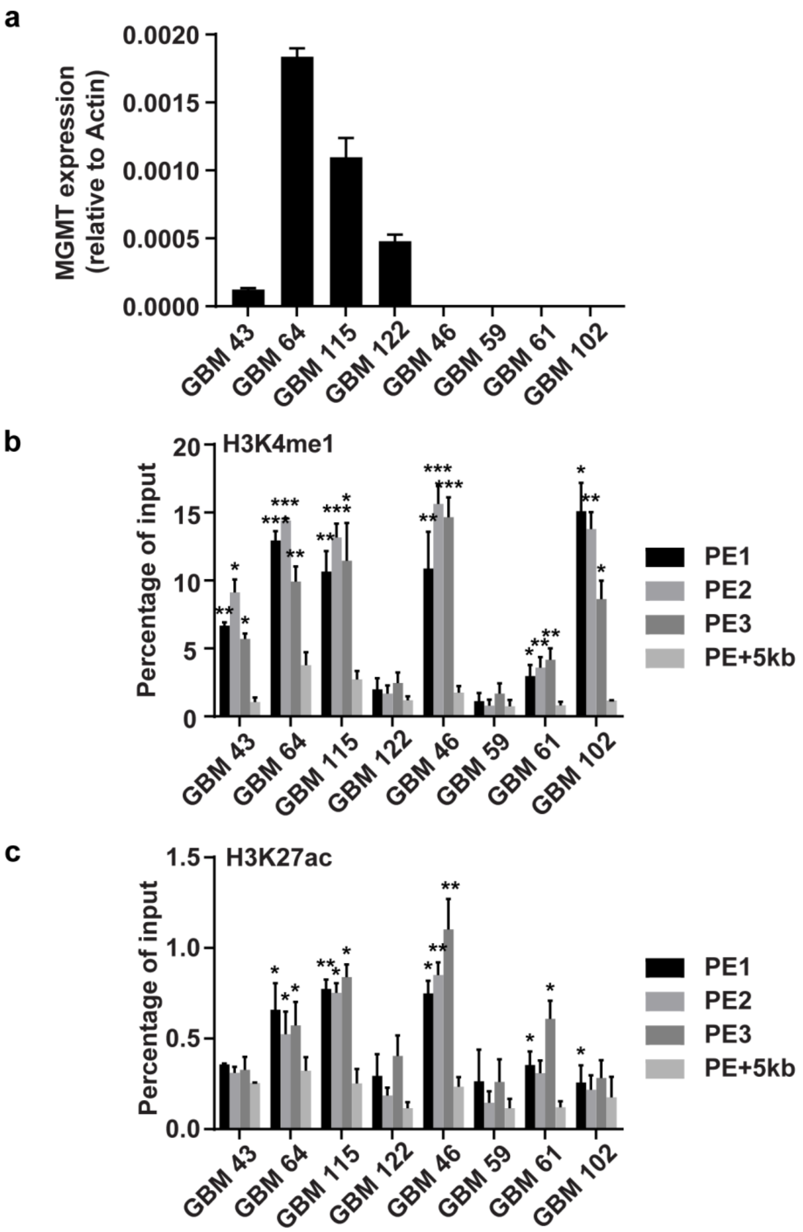

Supplementary Figure 3. MGMT expression and enhancer mark occupancy in promoter hypermethylated PDXs.

a. *MGMT* transcription levels in eight *MGMT* promoter methylated PDX tumors were analyzed by quantitative RT-PCR. MGMT expression was normalized to actin. **b-c.** H3K4me1 (**b**) and H3K27ac (**c**) occupancy at the putative K-M enhancer region was analyzed in eight PDX tumors by ChIP-qPCR with primers described in Figure 2. Reported values were derived from three biological repeats. (*p<0.05, **p<0.01, ***p<0.001, student's *t* test)

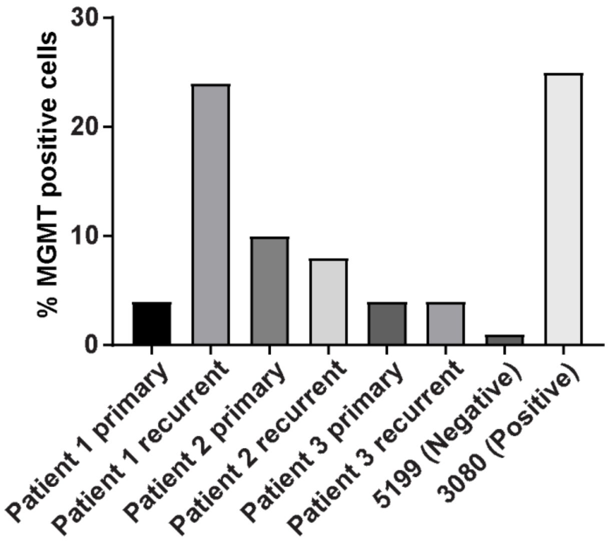

Supplementary Figure 4. MGMT expression in patient samples.

MGMT expression level in each patient sample was analyzed by immunofluorescence. One hundred cells are chosen randomly from each slide for analysis. MGMT expression level in each nucleus was analyzed based on the MGMT intensity (Intensity= average intensity × nucleus size, nucleus size is determined by DAPI staining). The cutoff line for MGMT positive staining was set based on the assumption that most cells in the 5199 line do not express MGMT.

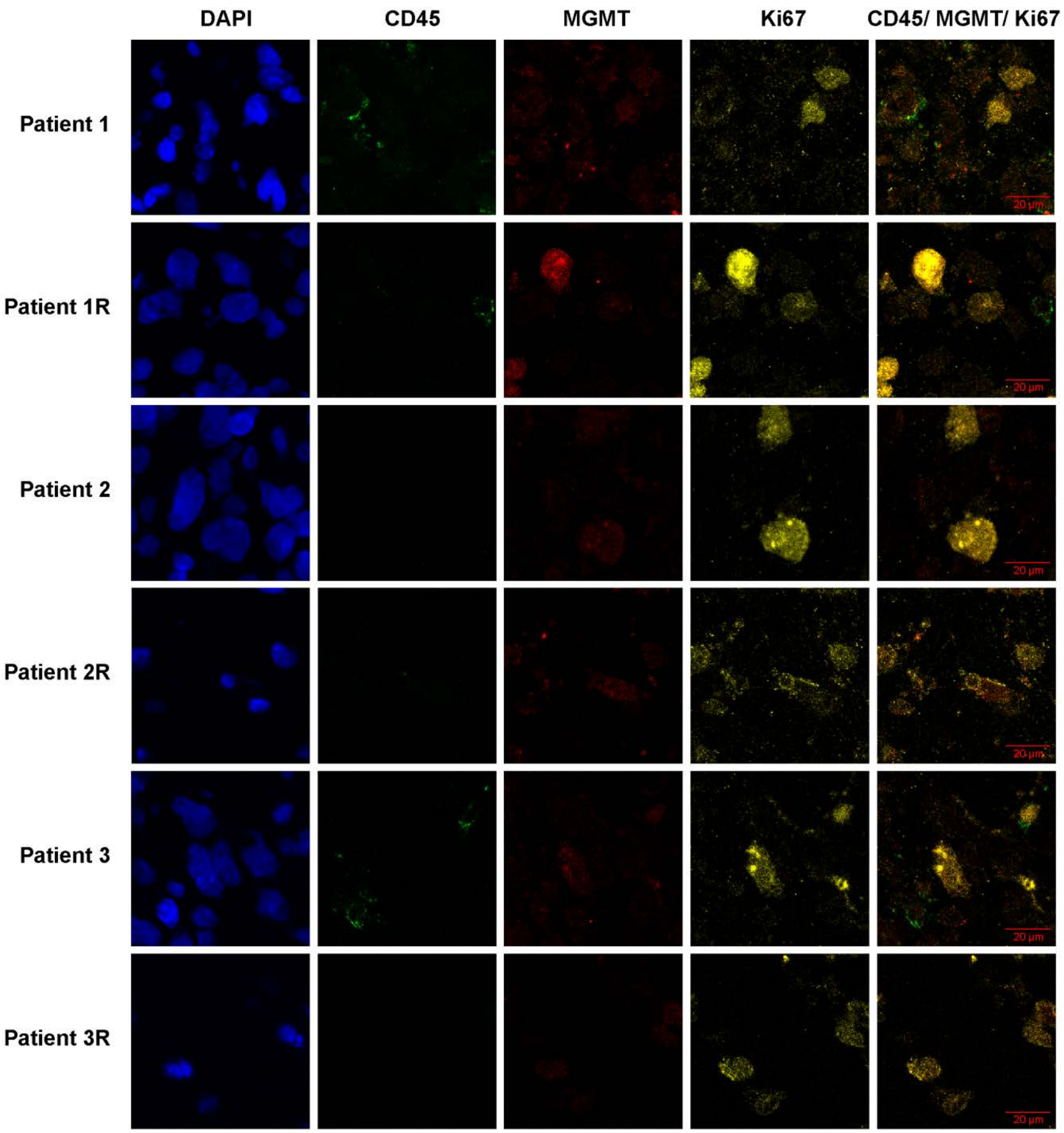

Supplementary Figure 5. CD45, Ki67, and MGMT triple immunofluorescence staining on GBM patient samples.

Representative images showing CD45, MGMT and Ki67 co-localization in tissue sections from primary and recurrent tumor obtained from patients #1, #2, and #3. Multicolor immunofluorescence was performed using antibodies against CD45, MGMT and Ki67.

a

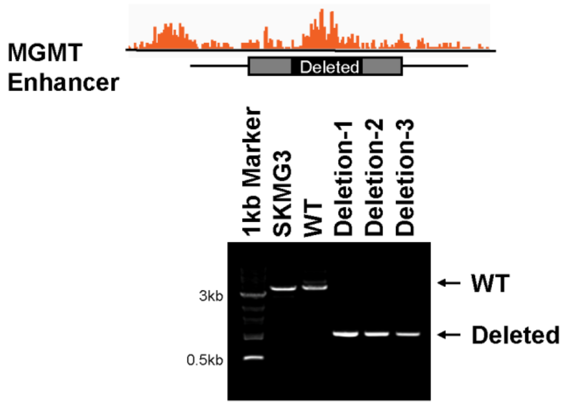

b

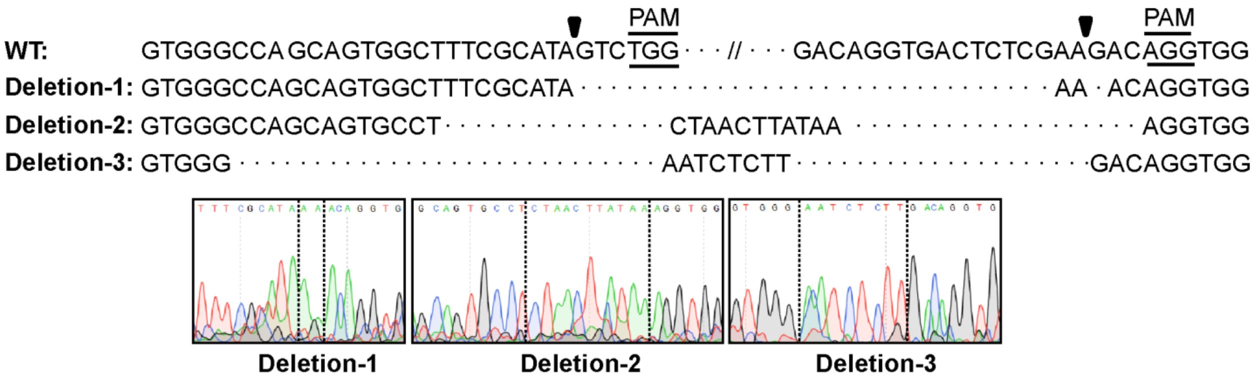

Supplementary Figure 6. PCR confirmation and Sanger sequencing for enhancer deleted clones.

a. Gel image showing successful deletion of a 3.3kb fragment in SKMG3 K-M enhancer deleted clones. One pair of primers spanning the sequence to be deleted was used for PCR assay. **b.** A successful deletion of K-M enhancer in three SKMG3 clones was confirmed by Sanger sequencing. The location of the PAM sequence (underlined), the predicted cutting site (marked by arrow head) and the deletion patterns detected by Sanger sequencing of all K-M enhancer deletion clones were aligned on the top panel. The raw Sanger sequencing plot is displayed in the lower panel.

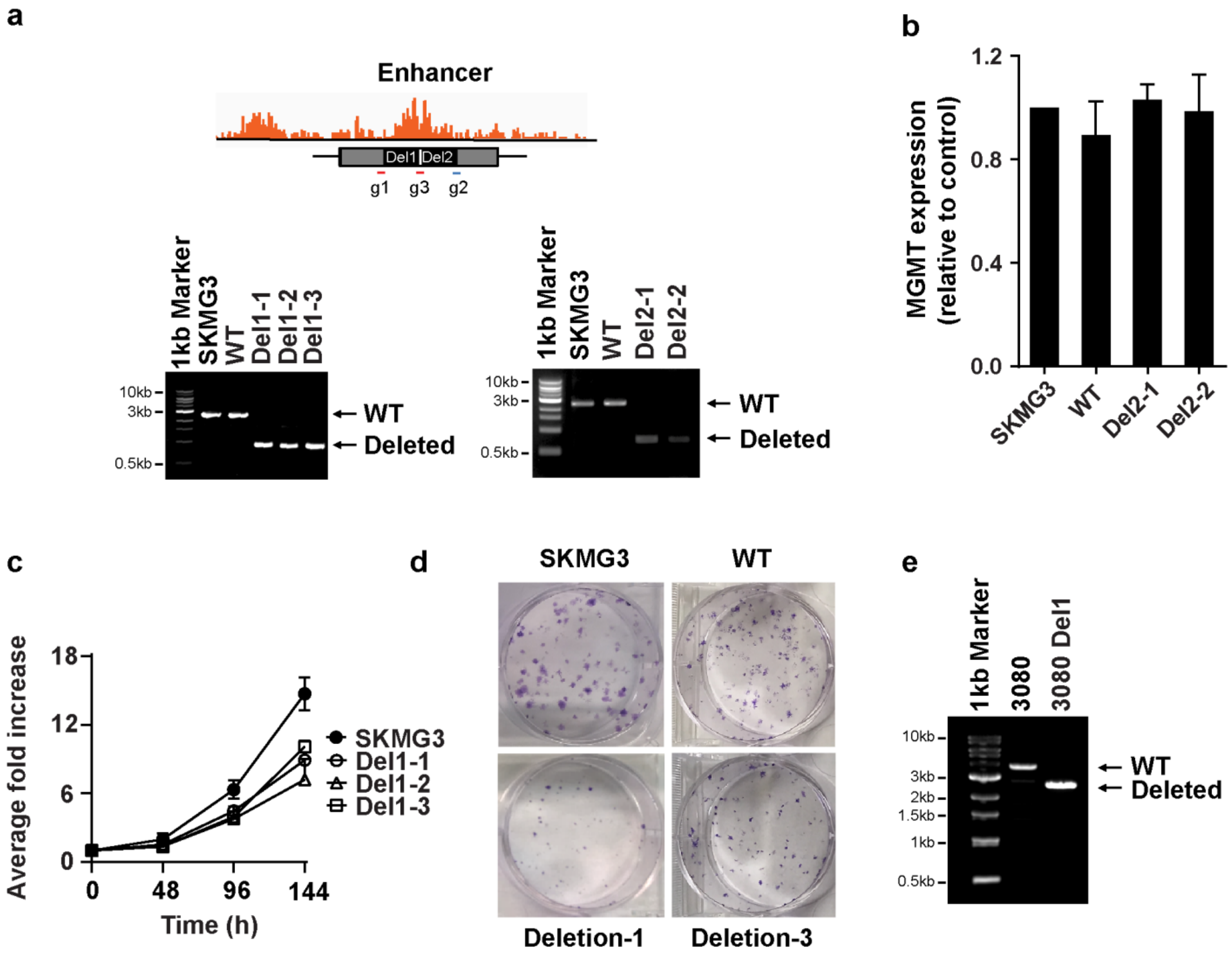

Supplementary Figure 7. PCR confirmation and MGMT expression of small deletion clones.

a. Gel image showing successful deletion of two DNA fragments in SKMG3 cells. One pair of primers spanning the sequence to be deleted was used for PCR assay. The regions to be deleted were indicated in top panel. Two pairs of primers, one spanning Del1 region another one spanning Del2 region, were used for PCR. **b.** MGMT expression in SKMG3 parental line, wild type clone and Del2 deleted clones were analyzed by quantitative RT-PCR. MGMT expression level was normalized to actin and subsequently calculated as fold change relative to SKMG3 parental line. **c.** The proliferation rate of SKMG3 parental cells and Del1 deleted SKMG3 clones were tested for proliferation using cell titer blue assay. The growth rate for each cell was normalized to Day 0 control. **d.** Images showing the clone size of SKMG3 parental cells, wild type clone and two deletion clones under DMSO treatment. The smaller clone size of enhancer deleted clones indicates impaired proliferation. **e.** Gel image showing successful deletion of Del1 region in 3080 cells.

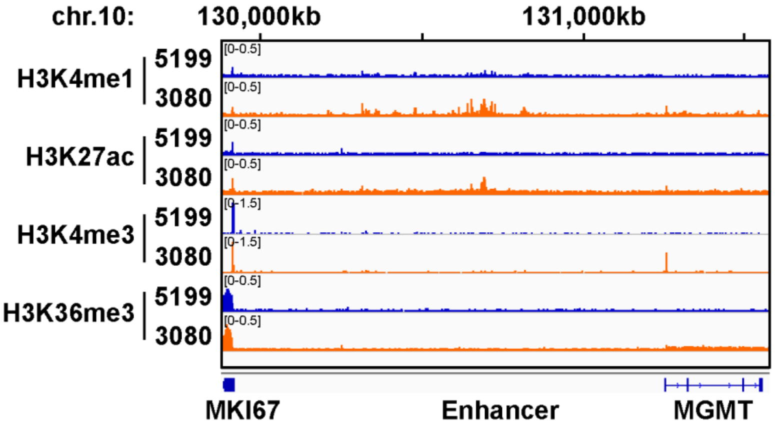

Supplementary Figure 8. Genomic location of *MKI67* gene.

An IGV snapshot showing H3K4me1, H3K27ac, H3K4me3 and H3K36me3 read density on *MKI67* gene, K-M enhancer and *MGMT* gene in 5199 and 3080 PDX tumors.

Supplementary Table 1. The number of peaks in the ChIP-seq data sets.

	**5199 line**	**3080 line**
**H3K4me1**	104389	110731
**H3K27ac**	55253	57347
**H3K4me3**	42720	38546
**H3K9ac**	61562	58607
**H3K9me3**	19414	17793
**H3K36me3**	110976	104009

Supplementary Table 2. Primers and TaqMan probe sequences used for MS-PCR and qPCR.

Primer name	Sequence (5'-3')	Assay
Actin-F	AGAGCTACGAGCTGCCTGAC	RT-PCR
Actin-R	AGCACTGTGTTGGCGTACAG	RT-PCR
MGMT-F	GCTGCGGTTCTCGGAGGTC	RT-PCR
MGMT-R	CTGCCAGGGCTGCTAATTGC	RT-PCR
MS-M-F	TTTCGACGTTCGTAGGTTTTCGC	MS-PCR
MS-M-R	GCACTCTTCCGAAAACGAAACG	MS-PCR
MS-U-F	TTTGTGTTTTGATGTTTTGTAGGTTTTTGT	MS-PCR
MS-U-R	AACTCCACACTCTTCCAAAAACAAAACA	MS-PCR
PE1-F	TACCAGGAATGATGTGGGGTTA	ChIP-qPCR
PE1-R	GGCATCTTCCCCTTCACAT	ChIP-qPCR
PE2-F	TGAACATGGCTTTTCAAAGGA	ChIP-qPCR
PE2-R	CAGCATTCCATGGTTGATGT	ChIP-qPCR
PE3-F	CCATGCAGGCATTTCTACCT	ChIP-qPCR
PE3-R	GAGCTCGCAGGAGCAACTAC	ChIP-qPCR
PE+5kb-F	TGCAAAATTTATTTGGGAGAAAA	ChIP-qPCR
PE+5kb-R	AGCTGCCATATAGAAAATTGGTC	ChIP-qPCR
Digestion test primer	CCTTTTGATAAATGCTTTCTGGA	3C
Anchor primer	ATAGCCTAATCTGCCAAACGA	3C
TaqMan Probe	FAM-CATGTCTTAAGCCACATACATTTGGTTCCT-TAMRA	3C
3C F1 primer	CCTTTCAGGCTTTATGGCACC	3C
3C F2 primer	TTGAATCATCTCTGGTTCTGTTT	3C
3C F3 primer	TCATCTTCAAATACTACTATGCCACTT	3C
3C F4 primer	TCAGTCTTTGCATCCCCTTTT	3C
3C F5 primer	GCCTTCTCACCCAGTACACAA	3C
3C F6 primer	GGCTGGCCTTGTACTCTCT	3C
MGMT TSS F	GGGAGAGAGAGAGAAAGATCTCC	3C
MGMT TSS R	GAGGCAGGGATGTCCATAAA	3C
P1	AAAATCACAGCACCGCACAG	K-M deletion confirmation-F
P2	TTGCAGTCTGGCTTCCTCAA	K-M deletion confirmation-R
Del1-P1	AAAATCACAGCACCGCACAG	Del1 deletion confirmation-F
Del1-P2	CTCGCAGGAGCAACTACT	Del1 deletion confirmation-R
Del2-P1	TGACAAAGAAACCCATCAGT	Del2 deletion confirmation-F
Del2-P2	TTTGACCTATGGGAGAAGTAGA	Del2 deletion confirmation-R

Supplementary Table 3. Guide RNA sequences for CRISPR-dCas9 based P300 targeting assay and CRISPR-Cas9 based genomic deletion assays.

**guide RNA names**	**Sequence**	**Used for**
g1	GCAGTGGCTTTCGCATAGTC	Deletion
g2	GACAGGTGACTCTCGAAGAC	Deletion
g3	TTACAAGTACTTACTCGCAT	Deletion/Targeting
T1	CAGCTCCTAGCTGAGTGTAA	Targeting
T2	CTAACGTGCTCAGAAGATGG	Targeting
T3	CCGATTTCTAAGTGCACAAA	Targeting
T4	GACTCCGTCGTCCTGCCACG	Targeting
