# Supplementary material for: A novel enhancer regulates MGMT expression and promotes temozolomide resistance in glioblastoma
Source: Nat Commun. 2018 Jul 27;9:2949. doi: 10.1038/s41467-018-05373-4 (PMC6063898; doi:10.1038/s41467-018-05373-4)
Supplement: Supplementary file 3 — Description of Additional Supplementary Files [file 41467_2018_5373_MOESM3_ESM.pdf]

Description of Additional Supplementary Files

File Name: Supplementary Data 1
Description: The original list of Group-1 putative enhancers. The genomic location of each putative enhancer in Group-1 cluster, shown in Figure 1, was listed. The H3K4me1 and H3K27ac change on each putative enhancer locus was also listed together with the calculation of significance. The mRNA ID for the RNA transcript of the gene closest to each putative enhancer was listed in column I. The fold change of expression for each transcript was calculated using the following formula: RNA-seq reads in 3080 line/RNA-seq reads in 5199 line.
